# Activation of Steroid and Xenobiotic Receptor (SXR, NR1I2) and Its Orthologs in Laboratory, Toxicologic, and Genome Model Species

**DOI:** 10.1289/ehp.10853

**Published:** 2008-03-12

**Authors:** Matthew R. Milnes, Adriana Garcia, Emily Grossman, Felix Grün, Jason Shiotsugu, Michelle M. Tabb, Yukio Kawashima, Yoshinao Katsu, Hajime Watanabe, Taisen Iguchi, Bruce Blumberg

**Affiliations:** 1 Department of Developmental and Cell Biology, University of California, Irvine, California, USA; 2 Japan NUS Co. Ltd., Tokyo, Japan; 3 Okazaki Institute for Integrative Bioscience, National Institute for Basic Biology, National Institutes of Natural Sciences, Okazaki, Japan

**Keywords:** endocrine disruption, metabolism, pesticides, phthalates, PXR, SXR, xenobiotics

## Abstract

**Background:**

Nuclear receptor subfamily 1, group I, member 2 (NR1I2), commonly known as steroid and xenobiotic receptor (SXR) in humans, is a key ligand-dependent transcription factor responsible for the regulation of xenobiotic, steroid, and bile acid metabolism. The ligand-binding domain is principally responsible for species-specific activation of NR1I2 in response to xenobiotic exposure.

**Objectives:**

Our objective in this study was to create a common framework for screening NR1I2 orthologs from a variety of model species against environmentally relevant xenobiotics and to evaluate the results in light of using these species as predictors of xenobiotic disposition and for assessment of environmental health risk.

**Methods:**

Sixteen chimeric fusion plasmid vectors expressing the Gal4 DNA-binding domain and species-specific NR1I2 ligand-binding domain were screened for activation against a spectrum of 27 xenobiotic compounds using a standardized cotransfection receptor activation assay.

**Results:**

NR1I2 orthologs were activated by various ligands in a dose-dependent manner. Closely related species show broadly similar patterns of activation; however, considerable variation to individual compounds exists, even among species varying in only a few amino acid residues.

**Conclusions:**

Interspecies variation in NR1I2 activation by various ligands can be screened through the use of *in vitro* NR1I2 activation assays and should be taken into account when choosing appropriate animal models for assessing environmental health risk.

The ability of a xenobiotic substance to induce protection against subsequent exposure and also to induce protection against exposure to other potentially toxic compounds was first described more than 30 years ago by Hans Selye (1971). It was quickly realized that such “catatoxic” compounds led to an increase in hepatic cytochrome P450 (CYP) enzyme activity (Einarsson and Gustafsson 1973) and that the substrates of the activated enzymes were relatively nonspecific. In 1998, activation of human CYP3A4 was shown to be primarily mediated by nuclear receptor subfamily 1, group I, member 2 [NR1I2; GenBank accession no. AY091855; National Center for Biotechnology Information (NCBI) 2007b]. For purposes of clarification, we use the trivial names of NR1I2 orthologs associated with specific taxonomic groups. This receptor is commonly known as the steroid and xenobiotic receptor (SXR) in primates (Blumberg et al. 1998), pregnane X receptor (PXR) in nonprimate mammals (Kliewer et al. 1998; Lehmann et al. 1998), chicken X receptor (CXR) in birds (Moore et al. 2002), and benzoate X receptor (BXR) in amphibians (Grün et al. 2002). It is now well established that the most prevalent CYP enzymes in the liver, members of the CYP3A and 2B subfamilies, along with a host of conjugating enzymes and ATP binding cassette (ABC) family membrane transport proteins, are under direct transcriptional regulation by NR1I2 (Xie et al. 2000b, 2004).

Through the action of its target genes, NR1I2 is a key regulator of bile salt, steroid hormone, and xenobiotic metabolism and excretion (Kliewer et al. 2002). NR1I2 is a member of the nuclear hormone receptor superfamily, which also includes sex steroid receptors, thyroid receptor, and other orphan receptors such as constitutive androstane receptor (CAR, NR1I3). The term “orphan receptor” has been given to a number of transcription factors that are related to nuclear receptors but for which a definitive endogenous ligand was not initially identified. Ligand-dependent activation of NR1I2 is mediated by steroid hormones, dietary compounds (e.g., phytoestrogens), vitamins E and K, medicinal herbs, xenobiotics, and approximately 50% of prescription drugs (reviewed by Dussault and Forman 2002; Kretschmer and Baldwin 2005). These ligands are extremely varied in chemical structure and application, and some have been shown to activate or antagonize NR1I2 orthologs in a species-specific manner (e.g., rifampicin, coumestrol, highly chlorinated polychlorinated biphenyls) (Blumberg et al. 1998; Jones et al. 2000; Tabb et al. 2004).

Development of the so-called humanized mouse was an important step in understanding the pharmacology of xenobiotic metabolism (Xie et al. 2000a). This animal is deficient in the rodent NR1I2 ortholog, PXR, and transgenic for human SXR expression in the liver. This model demonstrates convincingly that NR1I2 is the key regulator of CYP3A expression and that selective activation of target genes in response to species-specific activators depends on the ligand-binding domain (LBD) of this receptor, rather than on the DNA-binding domain (DBD) or target DNA-binding elements. The primary sequence of the LBD for NR1I2 varies greatly across species. The sequence similarity can be as low as 75% between mammalian NR1I2 orthologs and as low as 49% when comparing the chicken ortholog, CXR, to human SXR (Moore et al. 2002). A fundamental assumption made when using the results of model animal experiments to predict effects on humans or wildlife is that uptake and metabolism of the compound as well as the biochemistry and endocrinology of the organism is similar between the model species and species of concern. In some cases, the response of a model species to chemical exposure is reasonably predictive of the effects on humans. In other cases, the connection is more uncertain, and the ability to predict risk is not reliable. Understanding how the xenobiotic response differs among species is essential to developing high-quality models and characterizations of risk from chemical exposure.

The goal of this study was to screen a wide variety of xenobiotic compounds for interaction with NR1I2 orthologs within a common system, thus providing the framework for understanding the metabolism of xenobiotics in different model species. To compare responses correlating to interspecies variation in the LBD of NR1I2, we used an *in vitro* luciferase reporter assay driven by yeast Gal4 DBD-NR1I2 LBD fusion plasmids. Advantages of this system are that it eliminates the need to clone each species’ bona fide response and is insensitive to induction by endogenous receptors. One disadvantage of this system is that it is insensitive to interspecies variation in activation function-1 (AF-1) region coregulator recruitment; however, the availability of coregulators in any *in vitro* system is not necessarily representative of the *in vivo* environment. A structurally diverse array of xenobiotics was chosen (Table 1) to represent a broad spectrum of chemical classes and applications that published data indicate are of considerable environmental and/or health concern. NR1I2 orthologs tested included commonly used laboratory, toxicologic, and/or genome model species. The results of these experiments have important implications for determining the appropriate use of animal models and for assessing whether we can reasonably rely on those models to predict results in other species, including humans.

## Materials and Methods

### Cloning of NR1I2 orthologs

LBD coding sequence has previously been reported in GenBank for NR1I2 orthologs in human (accession no. AY091855), dog (AF454670), rabbit (AF182217), rat (AF151377), mouse (NM010936), chicken (AF276753), *Xenopus laevis* BXRα (BC041187) and BXRβ (AF305201), and zebrafish (AF502918). Novel NR1I2 LBD sequences were cloned from Japanese macaque (*Macaca fuscata*), crab-eating macaque (*Macaca fascicularis*), marmoset (*Callithrix jacchus*), quail (*Coturnix japonica*), fathead minnow (*Pimephales promelas*), fugu (*Takifugu rubripes*), and medaka (*Oryzias latipes*). For novel sequences, optimized degenerate primers (forward 5′-AGAACTAGTG-GATCCGYGARGGNTGYAARGGNTTYT T and reverse 5′-GGTATCGATAAGCTTG-CYTGCATNARNACRTAYTCYTC) were used for polymerase chain reaction (PCR) amplification of a region extending from the first zinc finger of the DBD (C E G C K G F F) into the LBD (E E Y V L M Q A) for each species. We used nested primers derived from the amplified region and 3′-RACE (rapid amplification of cDNA ends) to obtain full LBD sequences beginning with the coding region corresponding to human SXR Met-107 from liver cDNA libraries from each species. Gal4-NR1I2 fusion constructs were created by subcloning the LBD into *Eco*RI and *Bam*HI sites of the vector pCMX-Gal4N (Blumberg et al. 1998) using *Exo*III-mediated ligation-independent cloning (Li and Evans 1997). The PCR products were directly sequenced, and we selected GAL4-NR1I2 LBD clones that matched each consensus sequence.

### Cell culture and luciferase reporter assays

COS7 cells were maintained in phenol-red–free Dulbecco’s minimal Eagle medium (DMEM) supplemented with 10% fetal bovine serum (FBS). Twenty-four hours before transfection, we seeded 96-well plates with 5 × 10^5^ cells per plate. Chimeric receptor plasmids were cotransfected along with the tk(MH100)x4luc and pCMX-β-galactosidase reporter plasmids using calcium-phosphate–mediated transfection (Grün et al. 2002; Tabb et al. 2004). All ligands were initially dissolved in dimethylsulfoxide (DMSO) and subsequently diluted in DMEM supplemented with 10% charcoal-resin stripped FBS with a final concentration of 0.5% DMSO. The final DMSO concentration was minimized according to the solubility limits of the test compounds and adjusted so that all treatments were carried out under the same conditions. No overt toxicity, as indicated by β-galactosidase activity, was observed relative to untreated controls. After 24 hr of ligand exposure, we assayed 50-μL aliquots of cell lysate for luciferase and β-galactosidase activity, as previously described (Grün et al. 2002). Luciferase activity is reported as fold activation relative to the vehicle control (0.5% DMSO) and normalized for β-galactosidase activity. Each combination of receptor and ligand was run in triplicate at three doses and repeated whenever the coefficient of variance exceeded 0.15. Positive control ligands were assigned based on previously published data or empirically determined upon cloning of the novel orthologs. We also ran a negative control consisting of vector lacking an NR1I2 LBD for each ligand to ensure luciferase activity was not promoted via LBD-independent pathways.

### Sequence alignment and phylogenetic analysis

Novel sequences were checked for similarity using blastn and blastp (NCBI 2007a) and submitted to GenBank (NCBI 2007b). We used ClustalX (Thompson et al. 1997) to align deduced amino acid LBD sequences and create an identity matrix. A neighbor-joining phylogenetic tree was constructed using the PHYLIP computer program (Felsenstein 1989) using NR1I3 as a closely related outgroup.

## Results

### NR1I2 ortholog sequences

**Figure 1:**
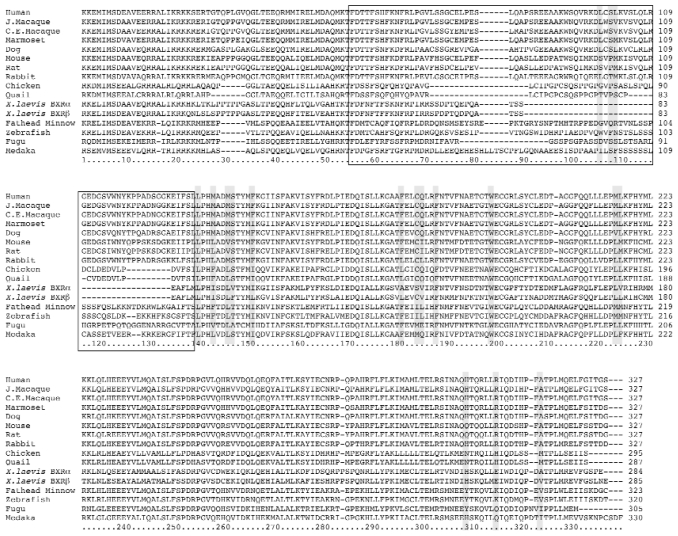
Alignment of amino acid sequences of NR1I2 ortholog LBDs. Shaded regions correspond to amino acid residues of the LBD that have been shown to interact with xenobiotic ligands in human SXR (Chrencik et al. 2005; Watkins et al. 2001, 2003). The boxed regions represent the helix 1–3 insert that distinguishes functionally divergent members of the NR1I subfamily (Moore et al. 2002). J. macaque, Japanese macaque; C.E. macaque, crab-eating macaque.

Comparison of NR1I2 ortholog LBD sequences (Figure 1) revealed a relatively high degree of similarity among mammalian orthologs compared to nonmammals. Human SXR amino acid residues that line the LBD and interact with various ligands (shaded) have been characterized by X-ray crystallography (Chrencik et al. 2005; Watkins et al. 2001, 2003). The corresponding residues appear to be highly conserved within mammals or are typically represented by functionally similar amino acid substitutions such as nonpolar valine, leucine, and methionine, or polar serine and threonine. Notable exceptions include the substitution of serine for leucine at position 105 in rodents and leucine or isoleucine for glutamine at position 184 in rabbits and rodents. When comparing nonmammalian NR1I2 orthologs, the least conserved region is the helix 1–3 insert, almost entirely absent in *Xenopus* BXRs, and highly variable among avian CXRs and fish orthologs. This region is thought to facilitate expansion of the ligand-binding pocket and distinguishes NR1I2 from functionally divergent members of the NRI1 family (Moore et al. 2002).

**Figure 2:**
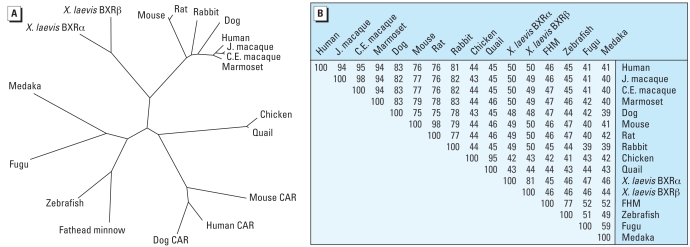
Nonrooted neighbor-joining tree of NR1I2 orthologs and mammalian NR1I3 ligand-binding domains (*A*), and the percent amino acid identities of NR1I2 otholog ligand-binding domains (*B*). Abbreviations: FHM, fathead minnow; J. macaque, Japanese macaque; C.E. macaque, crab-eating macaque.

Sequence similarity and associations observed in the neighbor-joining tree (Figure 2) are generally consistent with expected evolutionary relationships among the represented vertebrate classes and orders. These results also indicate that the nonmammalian orthologs are approximately equidistant from mammalian NR1I2 and NR1I3, consistent with the hypothesis that mammalian NR1I2 and NR1I3 resulted from a gene duplication of a nonmammalian ancestral ortholog (Handschin et al. 2004; Krasowski et al. 2005).

### Activation of NR1I2 othologs

All ligands (with the exception of organotins) were screened at concentrations of 0.5, 5, and 50 μM. Of the 27 xenobiotic compounds tested, phthalates and organochlorines were most effective at activating NR1I2 orthologs. Human SXR and murine PXR were readily activated by most phthalates at 5 μM (Table 2), whereas amphibian, zebrafish, fugu, and medaka orthologs were for the most part unaffected, even at the highest concentrations. At 50 μM, all organochlorines except octachlorostyrene induced a > 10-fold increase in luciferase activity relative to vehicle alone in many species (Table 3). Nonprimate mammalian, avian, and amphibian NR1I2 orthologs appeared most susceptible to organochlorine activation and exhibited moderate (4- to 10-fold) to high luciferase activity at 5 μM. With the exception of 2,4-dichlorophenol in medaka, most NR1I2 orthologs were completely insensitive to chlorinated phenols. The organotins, which are cytotoxic at micromolar concentrations, were tested at 1, 10, and 100 nM and failed to induce significant luciferase activity in any species (Table 4). Among the nonorganochlorine pesticides and industrial compounds, only the pyrethroid ester fenvalerate and the alkyl phenol surfactant 4*-tert*-octylphenol elicited significant luciferase activity at the 5-μM concentration.

## Discussion

Our results show significant variability across species in the capacity of xenobiotics to activate NR1I2 orthologs. Generally speaking, NR1I2 exhibits broad ligand specificity and regulates genes involved in hepatic metabolism of endogenous and xenobiotic compounds. As a result, this transcription factor presents unique challenges with regard to pharmacology and toxicology. For instance, the antibiotic rifampicin, a potent and selective activator of human SXR, can up-regulate hepatic metabolism of steroids to the extent that patients were incorrectly diagnosed with Cushing’s syndrome following overnight dexamethasone suppression tests (Kyriazopoulou and Vagenakis 1992). Furthermore, activation of SXR by one therapeutic compound can significantly alter the fate of another. Rifampicin and the herbal supplement St. John’s wort have both been shown to increase the clearance of the oral contraceptives ethinylestradiol and norethindrone (Barditch-Crovo et al. 1999; Hall et al. 2003).

Compounds that activated human SXR also activated SXR in nonhuman primates, but fold induction relative to the vehicle was typically lower in these species. In contrast, the nonprimate mammalian orthologs exhibited higher relative activation for many organochlorines and phthalates when compared to human SXR. Although fold induction of luciferase activity was variable across species for each ligand, all mammalian, avian, and amphibian orthologs appeared to be suitable qualitative models for predicting activation of human SXR with organochlorines. The two *Xenopus* BXRs had activation profiles similar to each other but were much less predictive of the human SXR response to phthalates compared to mammalian and avian orthologs. In contrast, responses among fish orthologs were so variable that few if any generalizations could be made. Medaka appeared to be insensitive to the vast majority of compounds tested, whereas the fathead minnow appears to be the most relevant model with regard to human SXR activation.

An important aspect of interpreting the toxicologic relevancy of these data is the comparison of the concentrations that elicit *in vitro* activation to predicted environmental exposure. One major limitation to analysis is that there is a relative paucity of data concerning the concentrations of chemicals in blood and other biological fluids in response to particular environmental concentrations. Another limitation to meaningful comparisons is that the method of reporting concentrations in biological and environmental samples is quite variable. For instance, the concentrations of many of the same organochlorine pesticides and phthalates used in this study have been measured in human breast milk as an indicator of neonatal exposure. The concentrations of organochlorines and other lipophilic compounds are reported as nanograms per gram lipid, and the major metabolites of phthalates, phthalate monoesters, are reported micrograms per liter. Assuming an average of 3–4% lipid in breast milk (Needham and Wang 2002), molar concentrations of organochlorines such as methoxychlor and *o,p*′-DDT were typically < 1 nM, whereas endosulfan and *p,p*′-DDE were in the 10–50 nM range (Damgaard et al. 2006; Shen et al. 2008). Although concentrations in breast milk are an order of magnitude lower than the minimum dose tested (500 nM), the daily intake of the infant should also be considered. Nanomolar to low micromolar concentrations were reported for phthalate monoesters in the breast milk of Danish and Finnish women (Main et al. 2006). Based on the concentrations in breast milk, infant body mass, and average milk consumption, the range of the estimated daily intake of some phthalates exceeded 50 μg/kg/day, the same dose used to up-regulate PXR-responsive genes with known ligands in laboratory mice (Xie et al. 2000a).

Although the toxicologic effects of activating NR1I2 are not completely understood, the metabolic pathways regulated by NR1I2 implicate it as a potential target for disrupting bile acid and steroid homeostasis (Zhai et al. 2007). Further complicating these interactions, xenobiotics that fail to activate this receptor may be more toxic than those that activate it and induce their own metabolism. NR1I2 mediates the metabolism of many drugs, and this metabolism can be induced to a very high level by chronic NR1I2 activation. Mice expressing a constitutively active form of human SXR (Alb-VPSXR) are almost completely resistant to the anesthetic effects of tribromoethanol and zoxaolamine, demonstrating this concept (Xie et al. 2000a).

The development of competitive binding and receptor activation assays allows one to estimate the potential for a xenobiotic compound to interact with a single receptor in any species. However, the ability to predict which chemicals will induce a characterized response *in vivo* at a particular dose, such as uterine proliferation via estrogen receptor (ER) activation, is much more complex. This matter is further complicated when considering exposure to a chemical that activates multiple transcription factors with different affinities. For instance, bisphenol A has an EC_50_ (half maximal concentration) of approximately 200 nM in ER luciferase reporter assays and the E-Screen cell proliferation assay (Gutendorf and Westendorf 2001). Our data indicate that bisphenol A activates NR1I2-dependent transcription at 50 μM and thus would induce its own metabolism at similar concentrations. Based on these data, one might predict an inverted U-shaped dose–response curve for bisphenol A *in vivo*, a phenomenon that has been repeatedly reported (for review, see vom Saal and Hughes 2005).

A significant difficulty in deriving an accurate risk assessment from laboratory experiments is the uncertainty about whether the underlying mechanisms of response to chemical exposure are universal. The use of *in vitro* or cell-based assays to guide and refine the development of *in vivo* models to screen compounds for NR1I2 activation is a useful tool to understand and/or prevent unintended xenobiotic interactions. Our results demonstrate species-specific differences in the ability of NR1I2 orthologs to activate transcription. This suggests that the metabolism, and presumably the physiological effects, of those ligands will also vary across species. Future work screening xenobiotics for toxicologic effects as well as drug–drug interactions should take these data into consideration.

## Figures and Tables

**Table 1:** Compounds tested for their ability to activate NR1I2 orthologs.

Compound	Classification	CAS no.	Supplier
4-*tert*-Octylphenol	Alkyl phenol	140-66-9	Wako Pure Chemical Industries, Osaka, Japan
Carbaryl	Carbamate	63-25-2	ChemService, West Chester, PA, USA
Pentachlorophenol	Chlorinated phenol	87-86-5	Wako Pure Chemical Industries, Osaka, Japan
2,4-Dichlorophenol	Chlorinated phenol	120-83-2	Tokyo Chemical Industry, Japan
Benzophenone	Industrial intermediate	119-61-9	ChemService, West Chester, PA, USA
4-Nitrotoluene	Industrial intermediate	99-99-0	Tokyo Chemical Industry, Tokyo, Japan
Chlordane	Organochlorine	57-74-9	ChemService, West Chester, PA, USA
Dieldrin	Organochlorine	60-57-1	ChemService, West Chester, PA, USA
*p,p*′-DDE	Organochlorine	72-55-9	ChemService, West Chester, PA, USA
Methoxychlor	Organochlorine	72-43-5	ChemService, West Chester, PA, USA
*o,p*′-DDT	Organochlorine	789-02-6	ChemService, West Chester, PA, USA
Toxaphene	Organochlorine	8001-35-2	ChemService, West Chester, PA, USA
Endosulfan	Organochlorine	115-29-7	ChemService, West Chester, PA, USA
Octachlorostyrene	Organohalogen	29082-74-4	ChemService, West Chester, PA, USA
Tributyl tin chloride	Organotin	1461-22-9	Sigma-Aldrich, St. Louis, MO, USA
Triphenyl tin chloride	Organotin	639-58-7	Sigma-Aldrich, St. Louis, MO, USA
Dibutyl phthalate	Phthalate	84-74-2	Wako Pure Chemical Industries, Osaka, Japan
Benzyl butyl phthalate	Phthalate	85-68-7	Wako Pure Chemical Industries, Osaka, Japan
Bis (2-ethylhexyl) phthalate	Phthalate	117-81-7	Wako Pure Chemical Industries, Osaka, Japan
Dicyclohexyl phthalate	Phthalate	84-61-7	Wako Pure Chemical Industries, Osaka, Japan
Diethyl phthalate	Phthalate	84-66-2	Kanto Chemical Company, Tokyo, Japan
Di-*n*-hexyl phthalate	Phthalate	84-75-3	Tokyo Chemical Industry, Tokyo, Japan
*n*-Dipentyl phthalate	Phthalate	131-18-0	Tokyo Chemical Industry, Tokyo, Japan
*n*-Dipropyl phthalate	Phthalate	131-16-8	Tokyo Chemical Industry, Tokyo, Japan
Bisphenol A	Plastic monomer	80-05-7	Tokyo Chemical Industry, Tokyo, Japan
Fenvalerate	Pyrethroid	51630-58-1	ChemService, West Chester, PA, USA
Amitrol	Triazine	61-82-5	ChemService, West Chester, PA, USA

CAS, Chemical Abstracts Service; *p,p*′-DDE, *p,p*′-dichlorodiphenyldichloroethylene; *o,p*′-DDT, *o,p*′ dichlorodiphenyl-trichloroethane.

**Table 2:** Species-specific activation of NR1I2 orthologs by phthalates.

Ligand	Exposure (μM)	Human	Japanese macaque	Crab-eating macaque	Marmoset	Dog	Mouse	Rat	Rabbit	Chicken	Quail	*X. laevis* BXR α	*X. laevis* BXRβ	FHM	Zebrafish	Fugu	Medaka
Diethyl phthalate	50	2.7	1.1	1.4	1.4	1.5	0.8	1.7	1.4	1.6	2.2	0.9	0.5	14.4	3.3	1.3	5.1
	5	1.0	0.6	0.7	0.8	1.0	0.5	0.9	0.7	0.9	1.0	0.7	1.0	2.4	0.7	1.1	3.3
	0.5	0.8	1.4	0.9	0.9	1.1	2.5	0.8	0.9	1.0	0.7	2.5	0.7	0.8	1.1	1.1	1.7
Benzyl butyl phthalate	50	11.4	4.8	3.6	4.4	12.0	23.6	18.9	12.0	7.1	10.1	3.1	2.0	8.5	1.8	1.6	1.4
	5	4.5	3.6	4.0	3.1	1.6	5.1	7.0	3.3	3.3	8.6	1.8	1.4	3.8	1.0	1.5	1.1
	0.5	1.0	1.4	1.3	1.0	1.4	1.1	1.1	0.9	1.2	1.7	1.0	0.7	1.0	0.7	0.8	1.0
Bis (2-ethylhexyl) phthalate	50	13.0	6.8	3.6	3.7	12.8	35.3	28.1	10.5	10.5	6.8	3.5	4.2	2.8	1.4	2.7	2.4
	5	10.7	12.4	9.8	5.8	3.5	33.6	30.0	6.7	6.3	6.3	1.9	3.8	1.7	1.1	3.7	2.3
	0.5	1.9	3.8	5.3	1.9	1.1	2.5	3.8	1.9	1.5	1.2	1.5	1.2	1.1	0.8	1.3	1.1
Dicyclohexyl phthalate	50	11.0	4.1	2.7	3.0	11.1	16.6	16.2	7.8	9.2	6.3	2.9	3.2	2.0	1.5	1.5	1.6
	5	10.1	5.5	5.8	5.7	3.7	12.1	10.4	3.0	5.8	7.9	1.9	2.9	1.6	2.7	2.5	1.3
	0.5	1.8	1.5	1.7	1.5	1.3	1.3	1.4	1.1	1.3	1.5	0.7	1.1	1.1	0.8	1.1	1.2
Dibutyl phthalate	50	10.9	4.2	4.5	4.2	9.9	19.6	19.5	10.5	7.9	5.4	2.3	1.0	14.1	2.7	1.6	1.9
	5	3.6	2.4	2.9	2.9	2.0	2.5	4.2	2.5	2.2	3.3	0.9	1.1	5.1	1.9	1.4	1.6
	0.5	2.5	1.4	1.1	1.0	1.0	0.7	1.1	1.1	1.1	0.9	1.1	1.1	0.9	0.8	1.0	1.0
*n*-Dipentyl phthalate	50	10.7	2.8	3.6	4.6	4.8	15.3	22.6	6.5	7.8	7.2	3.4	1.8	7.8	1.7	1.8	2.0
	5	2.6	2.9	4.0	4.1	0.6	4.2	5.9	2.2	2.9	7.5	3.4	1.4	1.6	0.8	2.4	1.5
	0.5	1.8	2.1	1.6	2.2	0.7	1.0	1.2	1.0	1.0	1.7	1.5	1.0	1.7	1.2	1.1	2.1
*n*-Dipropyl phthalate	50	8.1	4.0	4.6	6.3	2.0	8.8	12.3	4.4	6.8	5.7	5.0	1.0	36.2	6.2	2.3	5.7
	5	2.5	1.3	1.9	2.8	0.4	1.5	2.2	1.4	1.4	2.5	3.1	1.3	13.6	4.0	1.2	5.5
	0.5	2.2	0.7	1.0	1.4	0.4	1.0	1.1	0.7	0.9	1.1	1.1	0.8	2.4	0.6	1.3	2.3
Di-*n*-hexyl phthalate	50	10.6	3.7	3.7	5.1	3.4	20.1	21.4	4.9	9.5	10.5	4.3	2.5	6.4	1.6	2.4	1.5
	5	3.9	3.1	5.6	4.8	0.8	4.7	7.4	3.2	2.2	6.9	2.2	1.7	2.2	0.9	2.7	1.2
	0.5	1.2	1.0	1.8	1.9	0.5	0.9	1.1	0.7	1.0	1.5	2.0	1.1	1.1	0.7	1.5	1.4
Positive control^a^	50	22.8	5.9	7.3	14.6	17.8	68.6	41.3	10.2	17.7	22.8	32.1	94.0	—	—	5.8	12.8
	5	16.3	4.3	5.0	7.7	2.8	64.2	39.8	3.1	5.7	14.7	22.7	49.8	34.1	6.2	4.9	8.0
	0.5	3.7	1.6	2.2	2.6	1.4	36.7	26.6	1.0	1.0	4.4	10.9	8.8	24.2	3.0	2.4	2.6

FHM, fathead minnow. Values represent fold induction of luciferase activity (normalized for β-galactosidase activity) relative to DMSO treatment.

a Positive controls were as follows: rifampicin for human, macaque, dog, marmoset, rabbit, and fugu; pregnenolone 16α-carbonitrile for mouse and rat; 5β-3,20 pregnane dione for chicken and quail; *p*-hydroxy benzoic acid butyl ester for *Xenopus laevis BXR*α *and BXR*β*,* and medaka; and clotrimazole (cytotoxic at 50 μM) for FHM and zebrafish.

**Table 3:** Species-specific activation of NR1I2 orthologs by organochlorines and phenols.

Ligand	Exposure (μM)	Human	Japanese macaque	Crab-eating macaque	Marmoset	Dog	Mouse	Rat	Rabbit	Chicken	Quail	*X. laevis* BXRα	*X. laevis* BXRβ	FHM	Zebrafish	Fugu	Medaka
Chlordane	50	5.7	3.1	4.4	7.8	21.3	20.7	12.0	12.1	9.5	7.5	17.9	7.2	14.4	4.5	2.7	0.5
	5	8.9	3.7	3.3	5.8	5.1	10.8	14.4	6.7	5.3	10.6	4.3	5.1	7.9	2.4	3.0	0.8
	0.5	2.3	1.1	1.4	2.6	1.4	2.2	2.7	2.6	1.5	4.0	1.2	1.6	1.8	0.9	1.1	0.7
*o,p*’-DDT	50	16.3	5.1	6.9	12.3	27.0	45.3	32.3	12.6	17.3	23.8	16.7	15.8	11.9	4.0	9.1	1.3
	5	6.2	1.6	2.1	4.1	1.0	3.0	3.4	1.6	3.6	7.4	2.0	2.6	2.4	1.3	1.6	1.1
	0.5	1.3	1.1	1.1	1.0	0.8	1.1	0.6	0.9	1.2	1.3	1.1	1.2	1.1	1.0	1.3	1.2
*p,p*′-DDE	50	15.4	2.8	8.5	12.0	5.3	8.6	9.6	3.8	12.6	7.4	5.4	8.6	1.8	1.1	4.3	1.1
	5	2.1	1.2	1.4	2.2	0.7	1.2	1.1	1.0	1.7	1.6	1.4	1.7	1.4	0.6	1.1	1.1
	0.5	1.2	0.8	0.8	1.2	0.9	1.3	0.7	0.9	1.1	1.0	0.9	1.3	1.0	0.9	0.9	1.1
Dieldrin	50	11.0	3.4	5.9	11.6	9.8	33.0	25.7	16.3	10.4	13.8	9.3	4.8	5.6	1.6	2.8	0.2
	5	7.0	1.3	3.8	5.7	2.8	8.2	7.4	11.6	2.7	9.0	2.5	3.6	2.1	1.0	1.7	0.3
	0.5	2.0	1.1	1.3	3.3	1.0	1.5	1.6	2.2	1.4	3.4	1.3	1.8	2.2	1.0	1.0	0.5
Endosulfan	50	8.5	4.6	4.7	14.6	17.5	50.7	29.9	16.8	17.8	15.4	18.0	9.9	7.1	3.5	2.8	0.3
	5	4.7	2.6	3.1	5.6	1.3	7.0	9.1	8.6	3.3	7.8	1.9	3.0	2.5	1.0	1.4	0.3
	0.5	1.5	1.2	1.5	2.3	2.4	1.7	1.5	1.7	1.1	2.4	1.1	1.3	1.8	1.0	1.1	0.8
Methoxychlor	50	22.3	4.1	10.6	21.8	12.5	110.0	57.9	16.1	24.8	20.8	11.2	9.4	7.1	3.4	7.1	0.6
	5	3.9	1.5	2.4	3.9	1.0	16.5	29.8	2.5	3.5	5.2	1.7	2.5	2.0	1.4	1.3	1.0
	0.5	1.2	1.2	0.7	1.5	0.8	1.3	1.0	0.9	1.2	2.0	1.1	1.4	1.4	1.2	0.9	1.4
Octachlorostyrene	50	8.5	3.1	2.4	2.9	3.5	5.2	6.6	2.1	3.7	3.9	2.7	1.0	1.2	0.9	1.3	1.3
	5	1.3	1.0	1.1	1.2	1.4	1.2	1.4	0.9	1.1	2.3	0.7	0.7	1.9	0.6	1.3	1.2
	0.5	1.4	0.9	0.8	0.9	0.8	0.6	0.9	0.9	1.0	1.4	0.6	0.6	1.7	1.1	1.0	1.4
Toxaphene	50	10.9	5.1	6.5	16.3	41.9	38.2	22.4	16.3	16.0	25.8	21.5	12.5	19.8	5.5	2.8	0.8
	5	8.5	4.1	4.8	10.7	7.5	17.2	17.8	6.3	8.0	14.0	5.1	8.3	14.9	3.1	3.3	0.8
	0.5	1.9	1.1	1.3	2.6	0.7	2.3	1.9	0.9	1.7	4.7	1.3	1.8	3.7	1.2	1.1	1.0
2,4-Dichlorophenol	50	1.0	0.6	0.9	1.3	0.4	1.3	0.9	0.9	1.1	0.8	2.5	0.7	0.9	0.6	0.8	2.7
	5	1.1	0.8	1.1	1.4	0.3	1.2	0.9	0.7	1.0	1.0	1.4	0.8	1.8	0.7	1.0	5.0
	0.5	0.9	0.8	1.2	1.4	0.5	0.7	1.0	0.7	1.1	1.3	0.9	0.9	1.7	1.0	1.1	5.7
Pentachlorophenol	50	0.8	0.9	1.4	1.3	0.3	1.5	1.0	0.7	0.7	0.9	1.3	1.4	0.8	0.5	1.0	1.3
	5	1.7	0.9	1.5	1.2	0.4	3.1	1.0	0.6	0.8	1.0	2.3	1.1	1.0	0.8	1.2	1.1
	0.5	0.8	0.8	1.0	1.1	0.4	0.6	1.0	0.7	1.1	0.9	2.8	0.9	0.7	0.7	1.1	1.2

FHM, fathead minnow. Values represent fold induction of luciferase activity (normalized for β-galactosidase activity) relative to DMSO treatment.

**Table 4:** Species-specific activation of NR1I2 orthologs by selected nonorganochlorine pesticides, organotins, and industrial compounds.

Ligand	Exposure (μM)	Human	Japanese macaque	Crab-eating macaque	Marmoset	Dog	Mouse	Rat	Rabbit	Chicken	Quail	*X. laevis* BXRα	*X. laevis* BXRβ	FHM	Zebrafish	Fugu	Medaka
4-Nitrotoluene	50	2.1	0.6	1.1	1.5	0.4	1.6	1.2	0.9	1.3	1.1	2.3	0.9	1.0	0.9	1.4	6.6
	5	1.0	0.8	1.0	1.4	0.4	1.1	1.3	1.1	1.0	0.9	2.1	1.1	1.3	0.7	1.4	5.1
	0.5	0.8	1.2	1.0	1.7	0.7	0.8	1.0	2.0	1.6	1.0	1.1	1.1	1.3	0.7	1.8	2.0
4-*tert*- Octylphenol	50	15.9	3.5	4.0	7.3	12.8	13.4	20.4	7.5	8.7	7.7	12.5	5.2	17.1	4.6	3.3	6.0
	5	10.2	2.0	3.2	4.4	2.4	1.7	2.3	1.4	5.4	6.5	4.8	2.3	6.0	3.1	3.6	6.4
	0.5	1.3	1.0	1.5	1.6	0.5	0.7	1.0	1.0	1.4	2.6	1.1	1.2	1.4	0.9	1.5	6.7
Amitrol	50	0.7	1.0	0.8	1.1	0.3	2.2	1.0	0.5	1.0	1.3	1.7	0.8	0.9	0.7	1.1	4.2
	5	2.0	0.7	1.1	1.7	0.5	0.6	0.9	0.7	1.0	1.4	1.4	0.7	1.1	0.9	1.7	6.5
	0.5	4.3	2.3	1.6	2.2	0.9	1.3	1.3	2.6	1.5	4.6	3.0	1.2	3.5	1.0	1.5	6.3
Bisphenol A	50	11.0	3.8	3.7	5.4	1.0	3.0	2.5	4.8	9.2	6.3	6.0	5.3	2.8	2.3	4.9	3.5
	5	1.9	0.7	0.8	1.5	0.3	0.8	1.8	1.3	1.6	1.5	1.3	1.2	0.8	0.8	1.1	3.2
	0.5	0.9	0.7	0.8	1.1	0.4	0.9	1.0	0.7	1.1	1.2	1.2	0.8	0.8	0.8	1.6	2.1
Benzophenone	50	1.1	1.4	1.2	1.3	0.9	0.8	1.2	0.8	2.1	2.5	20.0	0.5	1.6	0.7	0.8	1.8
	5	0.9	1.1	0.8	0.9	1.9	0.9	0.8	0.7	1.1	1.3	7.9	0.7	1.1	0.8	1.0	1.5
	0.5	1.5	1.3	0.9	0.9	1.1	1.0	1.3	2.0	1.1	2.1	1.3	0.7	1.1	0.5	0.9	1.2
Carbaryl	50	2.3	0.9	1.9	3.0	1.8	1.8	1.2	2.3	1.5	2.1	1.7	1.6	2.0	1.4	1.2	1.7
	5	1.5	0.8	1.1	1.5	0.9	1.4	0.9	1.4	1.5	2.3	1.1	2.0	1.4	1.4	0.9	0.8
	0.5	2.8	1.5	2.1	2.6	1.2	2.3	2.0	1.9	1.9	3.9	1.6	2.3	2.7	1.3	1.4	0.8
Fenvalerate	50	13.7	6.6	10.4	19.8	60.7	65.9	41.7	32.8	23.5	23.3	11.6	11.8	4.0	6.2	8.4	1.0
	5	12.6	4.1	6.6	8.4	5.2	29.2	28.6	7.8	11.6	9.7	5.2	6.1	3.4	3.1	4.1	1.0
	0.5	2.0	0.9	1.8	2.0	1.0	1.6	1.9	1.6	1.6	1.6	1.2	1.3	1.4	0.9	1.0	1.0
Tributyl tin	100	2.0	0.8	0.9	0.7	1.3	1.4	0.6	1.4	1.3	0.9	0.7	1.8	0.6	0.7	0.5	0.9
chloride	10	0.9	1.0	0.8	0.7	1.2	1.0	0.6	0.7	1.3	1.3	0.8	1.2	0.6	0.7	0.8	0.9
	1	0.9	0.8	0.8	0.8	0.8	0.7	1.0	1.2	1.1	0.8	0.7	0.9	0.9	0.4	1.0	1.1
Triphenyl tin	100	0.5	1.0	0.8	1.3	1.1	0.6	0.7	2.7	1.4	0.9	0.9	1.0	0.8	2.0	1.7	0.9
chloride	10	1.1	0.7	0.9	0.9	1.6	0.3	1.4	1.0	0.9	0.8	0.6	1.2	1.3	0.9	1.9	1.1
	1	0.7	1.0	1.0	0.8	1.2	0.4	0.8	2.1	0.9	1.0	0.6	1.1	0.9	0.8	1.2	1.2

FHM, fathead minnow. Values represent fold induction of luciferase activity (normalized for β-galactosidase activity) relative to DMSO treatment.
